# Gaucher Cells or Pseudo-Gaucher Cells: That’s the Question

**DOI:** 10.4274/tjh.2014.0019

**Published:** 2014-12-05

**Authors:** Deniz Gören Şahin, Hava Üsküdar Teke, Mustafa Karagülle, Neslihan Andıç, Eren Gündüz, Serap Işıksoy, Olga Meltem Akay

**Affiliations:** 1 Eskişehir Osmangazi University Faculty of Medicine, Department of Hematology, Eskişehir, Turkey; 2 Eskişehir Osmangazi University Faculty of Medicine, Department of Pathology, Eskişehir, Turkey

**Keywords:** Crystalline inclusion bodies, Gaucher cells, Multiple myeloma

## TO THE EDITOR

Bone marrow cells with morphological characteristics similar to Gaucher cells and without cytoplasmic crystalline inclusions are rare. These Gaucher-like or pseudo-Gaucher cells can be seen in a variety of conditions such as acute lymphoblastic leukemia, multiple myeloma, myelodysplasia, Hodgkin’s disease, thalassemia, and disseminated mycobacterial infection [1,2,3,4,5,6,7,8]. Since the presence of these cells may obscure neoplastic cells in multiple myeloma and may lead to misdiagnosis, it is important for hematologists and hematopathologists to be aware of such a condition in order to make a prompt and accurate diagnosis. Herein we report a case of multiple myeloma in which the presence of plasma cells was missed on initial histological diagnosis.

**Figure 1:**
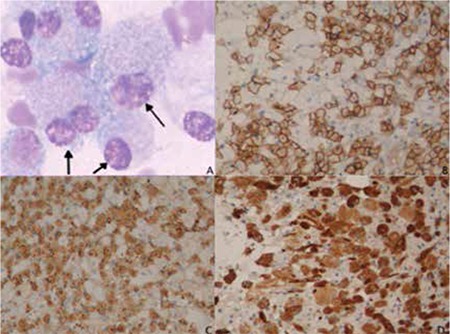
(A) Bone marrow aspirate smear (Giemsa 400x) showing pseudo-Gaucher cells with abundant cytoplasm, dense round deposits, and an eccentric pyknotic nucleus; immunohistochemical staining of bone marrow biopsy showed that plasma cells were positive for (B) CD38 and (C) kappa light chain; (D) the large cells were positive for CD68.

A 44-year-old female without history of any previous systemic disease presented with oliguria, easy fatigability, and breathlessness for 7 days. On examination she had crepitating rales, jugular venous congestion, abdominal distension, and pretibial edema. The complete blood count showed Hb of 67 g/L, WBC count of 3.4x109/L, and platelet count of 69x109/L. Erythrocyte sedimentation rate was 112 mm/h. Bone marrow aspirate and biopsy were performed. The bone marrow aspirate revealed numerous large cells with plentiful cytoplasm and a small eccentric nucleus. Scattered among these were plasma cells, which were obscured by sheets of Gaucher-like cells (Figure 1A). Immunohistochemical staining of bone marrow biopsy showed that plasma cells were positive for CD38 and kappa light chain (Figures 1B and 1C), and the large cells were positive for CD68 (Figure 1D). There were crystalline inclusion bodies within these cells, which were negative for smooth muscle actin, HHF-35, and keratin. The erythroid and myeloid series were normal. Serum electrophoresis revealed an M band. Skull X-ray showed lytic bone lesions. Taken together, a diagnosis of multiple myeloma associated with a prominent pseudo-Gaucher histiocytic response was made.

Gaucher-like cells have been described in various hematological disorders [1,2,3,4,5,6,7,8]. These cells are considered to be marrow macrophages seen in circumstances related to high cell turnover [9]. One striking feature is that pseudo-Gaucher cells cannot be distinguished from true Gaucher cells by routine hematoxylin-eosin staining. In order to differentiate them, iron staining should be performed. Gaucher cells show diffuse iron staining whereas pseudo-Gaucher cells do not. Electron microscopical features may also help distinguish pseudo-Gaucher cells from true Gaucher cells. On electron microscopy, pseudo-Gaucher cells do not contain typical tubular cytoplasmic inclusions, which are present in Gaucher cells. In addition, crystal-storing histiocytosis and sea blue histiocytosis should be considered in differential diagnosis. Macrophages with cytoplasmic crystalline inclusions are better regarded as crystal-storing histiocytes and this rare entity could be confused with Gaucher or pseudo-Gaucher cells [10]. Moreover, sea blue histiocytes should be kept in mind. However, these cells are heavily granulated with prominent vacuolation. We are reporting this case to increase the awareness among hematologists and hematopathologists of this rare association to avoid misdiagnosis. We also would like to highlight that the presence of pseudo-Gaucher cells in bone marrow should not be overlooked as they might be obscuring an underlying pathology. Awareness of possible associations, appropriate immunohistochemistry, and relevant additional investigations based on clinical findings are necessary for final diagnosis.

**Conflict of Interest Statement**

The authors of this paper have no conflicts of interest, including specific financial interests, relationships, and/or affiliations relevant to the subject matter or materials included.
